# Analysis of Air-Coupled Transducer-Based Elastic Waves Generation in CFRP Plates

**DOI:** 10.3390/s21217134

**Published:** 2021-10-27

**Authors:** Tomasz Wandowski, Damian Mindykowski, Pawel Kudela, Maciej Radzienski

**Affiliations:** Institute of Fluid–Flow Machinery, Polish Academy of Sciences, Fiszera 14 St., 80-231 Gdansk, Poland; dmindykowski@imp.gda.pl (D.M.); pk@imp.gda.pl (P.K.); mradzienski@imp.gda.pl (M.R.)

**Keywords:** air-coupled transducer, scanning laser doppler vibrometry, composite fiber-reinforced polymer

## Abstract

In this paper, the analysis of non-contact elastic waves generation in carbon fiber reinforced-polymer (CFRP) plate was conducted. Full non-contact elastic waves generation and sensing methods were also analyzed. Elastic waves generation was based on an air-coupled transducer (ACT) while waves sensing was based on a laser Doppler vibrometer. The excitation frequency was equal to 40 kHz. An optimal ACT slope angle for the generation of elastic waves mode was determined with the aid of dispersion curves calculated by using a semi-analytical model. Due to the stack sequence in the composite plate (unidirectional composite), ACT slope angles were different for waves generation in the direction along and across reinforcing fibers direction. Moreover, experimental verification of the optimal ACT slope angles was conducted. It was possible to generate A_0_ wave mode in the direction along and across the reinforcing fibers. Optimal angles determined using ACT were equal to 16° (along fibers) and 34° (across fibers). In the case of optimal angles, elastic waves amplitudes are almost two times higher than for the case of ACT oriented perpendicularly to the plate surface. Moreover, experimental results based on ACT showed that it was possible to generate the SH_0_ mode in the direction across the fiber for optimal angles equal to 10°. Finally, based on the A_0_ wave mode propagation, the process for localization of discontinuities was performed. Discontinuities in the form of additional mass simulating damage were investigated. A simple signal processing algorithm based on elastic wave energy was used for creating damage maps. Authors compared discontinuity localization for ACT oriented perpendicularly to the plate and at the optimal slope angle. The utilization of non-contact waves excitation at optimal ACT slope angles helped to focus the wave energy in the desired direction. Moreover, in this case, elastic waves with the highest amplitudes were generated.

## 1. Introduction

A carbon fiber-reinforced polymer (CFRP) is one of the most popular materials used in modern industry branches (including the aerospace and automotive industry). It is mainly due to its interesting material properties (good elastic properties and load resistance while keeping low mass [1,2]). Together with still growing interest in CFRP application for different structures, it is desired to be sure that any potential defects and discontinuities are detected early, thereby avoiding unwanted accidents or catastrophes [2]. There are many non-destructive testing (NDT) methods that are devoted to an inspection of structures [1,3], although, with various accuracy, it should be underlined that CFRP structures are highly anisotropic, which makes the process of their monitoring difficult and challenging [4]. A group of NDT methods that can be especially distinguished is ultrasonic testing (UT), due to its important advantages (possibility of fast and accurate inspection over the relatively big region while keeping the operation costs and safety of the inspection process at a low level).

Researchers are recently focused on the non-contact ultrasonic methods [5,6,7], which rely on non-contact generation and sensing of elastic waves propagating in the structure. However, the issue of efficient non-contact generation of acoustic waves (later converted into elastic waves due to interactions of the waves in the zone of air/solid interface) is still the subject of many works [6,7]. Due to a significant impedance mismatch between the air and solid, the acoustic waves are converted to elastic waves with low amplitudes compared to contact methods of elastic waves generation. The next problem is related to the fact that, for low frequencies bellow 100 kHz in thin films, for example, the velocity of the A_0_ mode in plastic/composite materials can become slower than the ultrasound velocity in air, and its propagation in films is accompanied only by an evanescent wave in the air [8]. In the case of very thin films (e.g., 150 µm thickness), the phase velocity of the A_0_ mode is slower than the ultrasound velocity in the air in the frequency range up to 300 kHz [9].

Hence, many researchers still prefer hybrid ultrasonic methods in their considerations, consisting of contact elastic waves generation and their non-contact detection [10,11,12,13]. The most popular way to generate elastic waves in contact way is the usage of piezoelectric ceramic transducer (PZT) [11,14,15]. Usually, small PZT discs with a diameter of ~10 mm [4,10] and a thickness of ~0.2 mm [14,15] are used. They generate elastic waves in the frequency range of ~100–200 kHz [10,11,12,13,14,15]. Besides simple PZT transducers, other types of transducers are utilized for this purpose (angle beam transducers [16,17,18], macro fibre composite actuators [19], or conventional ultrasonic probes [16]).

However, non-contact waves generation or sensing is still the field of interest for many researchers. In the case of non-contact waves generation laser sources, for example, pulsed lasers [7,20] are utilized. However, more popular are acoustic waves in the air which are then converted to elastic waves in structure. Air-coupled transducers (ACTs) [5,20,21] are utilized for this purpose. Typical excitation frequencies of ACTs used in various works are quite similar to these observed while using PZTs—dependent on the research, it can be equal to 100 kHz [22], 200 kHz [6,21], or even 1 MHz [5,7]. In terms of the facility used for non-contact sensing of elastic waves, propagating in the structure scanning laser Doppler vibrometry (SLDV) is utilized [7,11,23]. Some researchers use ACT not only for waves generation but also for sensing [5,6,22]. Additionally, optical microphone [21] or contact transducers [16,17,18,19] are also utilized for sensing.

Many works are focused on efficient defects detection, in the case of numerical and experimental data. These defects, such as delaminations [11,24,25,26] and low-velocity impact damage [6], are often considered in the case of the CFRP material, as these defects are often spotted in practice and, thus, their early detection is desired. Delaminations are often impact-induced [20] or simulated by thin Teflon film inserts between specific layers of a composite laminate [24]. Furthermore, defects such as holes [5,7,13] or cracks [21,27] are sometimes considered.

The choice of the excitation signal is important in experimental techniques. Most often sine modulated by the Hann window (toneburst signal), consisting of three [15,18] or five [10,11,13] sine cycles, is utilized. Different elastic wave modes are utilized for the detection of different damage types. The antisymmetric mode A_0_ was utilized for the detection of notches emanating from through-thickness holes [28], notch [29], disbonds in an elastic–viscoelastic (steel-rubber) bilayer [30], sub-surface delamination [31], and delamination in aluminum–CFRP samples [32]. In the case of the S_0_ mode, it was utilized for detection of notch [29,33], impact damage, teflon insert, and wrinkle damage in crossply composite plate [34]. The shear horizontal SH_0_ mode has a simple wave structure and a non-dispersive nature in metallic plates. This mode was also utilized for impact and teflon insert detection [34]. Moreover, the SH_0_ mode could be utilized for corrosion detection in pipelines because it is more sensitive to corrosion than the A_0_ mode [35]. Wavefield of SH_0_ mode is uniform through the thickness of the plate so it is expected to be equally sensitive to surface and interior defects [36]. Most of the analyzed structures are plate-like structures, with the thickness varying from ~1 mm [4,16] to ~4 mm [10,12,19]. However, few researchers analyzed structures with more complex shape. Authors of [22] performed an NDT inspection of complex stiffened wing-type structure, with a variable sheathing thickness of 2.4–5 mm and a thickness of the stiffeners equal to 2.4 mm. Besides the works concerning damage detection, some studies are focused on effective elastic waves propagation in CFRP structures.

Besides of experimental part of the research, its other relevant part is often the attempt to model investigated phenomena via a numerical way. Very popular is finite element method implemented in Abaqus [22,27], Comsol [17,26], or Ansys [26]. In turn, Leckey et al. [24,26] performed a comparison of different simulation programs (Abaqus, Ansys, Comsol, and custom-made numerical code based on elastodynamic finite integration technique) from the viewpoint of modelling guided waves propagation in the case of two different CFRP plate-like structures. Barski et al. [37] developed the stiffness matrix-based C++ code in order to check the impact of the plate fibers on the course of dispersion curves. Eremin et al. [4] validated the elastic properties of the CFRP structure via experimental way, comparing the results with initially known values of the properties (Young modulus, Kirchhoff modulus, and Poisson’s ratio). In order to determine dispersion curves, a semi-analytical finite element (SAFE) method based on classic finite elements through the thickness of a laminate was proposed in [38]. For this purpose, a semi-analytical spectral element (SASE) method based on spectral elements was also utilized [39]. Extraction of dispersion curves is very important in order to determine the optimal ACT slope/incident angle based on Snell law and phase velocity of an elastic wave [6,7,22].

The problem of an optimal ACT angle and the problem of ACT-based elastic waves generation in composite material is still not sufficiently analyzed. This was the motivation for the research presented in this paper and the continuation of research results presented in [40]. In fiber-reinforced composite materials, elastic wave propagation depends on fibers orientation; therefore, optimal ACT incidence angles depend on the direction of wave generation in relation to fiber orientation. We investigated the possibility of the generation of different elastic waves modes. Furthermore, the problem of the influence of ACT incidence angle on plate surface coverage by elastic waves with relatively high energies in order to perform discontinuities localization. A full non-contact elastic waves generation and sensing method was proposed. Moreover, studies were related to a very popular and cheap acoustic transducer with a relatively low frequency of 40 kHz. The effectiveness of this transducer in the discontinuity detection and localization in composite material was analyzed.

## 2. Air-Coupled Transducer-Based Elastic Waves Generation

**Figure 1:**
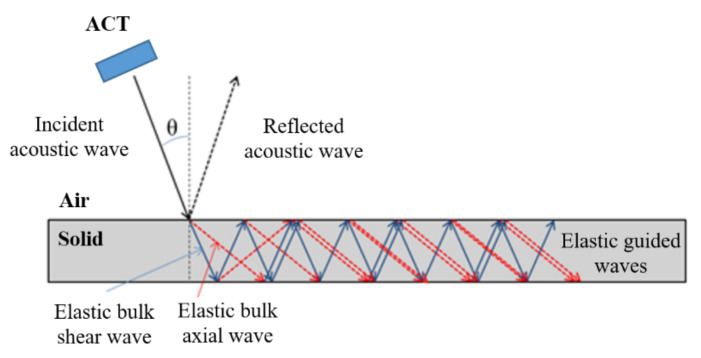
The conversion scheme of acoustic waves (AW) into elastic waves.

The air-coupled transducer generates the acoustic waves that propagate through the air. At the time instant at which the acoustic wave arrives to the solid, specimen is reflected as an acoustic wave from a specimen and transmitted to the specimen, where it converts to elastic bulk waves (longitudinal and shear). Due to the internal reflection of bulk waves in thin-walled structure, elastic guided waves are generated (Figure 1).

In this plate with parallel surfaces, elastic guided waves in the form of the shear horizontal SH mode and symmetric S and antisymmetric A Lamb waves modes could propagate.

An important factor related to the conversion of acoustic waves into elastic waves and the efficient utilization of ACT is the transducer slope/incident angle (θ). This angle is located between the transducer axis and line perpendicular to the plate surface (see Figure 1). Its proper adjustment strongly affects amplitudes of elastic wave modes propagating in the analyzed plate. The scheme of conversion of acoustic waves into elastic waves is depicted in Figure 1.

By knowing the sound velocity in the air at specific conditions and phase velocity of the certain elastic wave mode which is desired to be generated, the optimal ACT slope angle can be calculated based on the Snell law [23]:

(1) $$\theta_{opt}=sin^{-1}\left(\frac{c_{air}}{c_{p}}\right),$$

where $c_{air}$—the sound velocity in the air (~343 [m/s] assuming the temperature of 20 °C and the pressure of 1 bar), and $c_{p}$—the phase velocity of the selected wave mode [m/s]. The phase velocity of the elastic wave mode depends on the material properties. Moreover, in the case of Lamb wave modes which are dispersive, its phase velocity depends on the frequency of excitation and plate thickness. The number of elastic wave modes propagating in the structure is also related to the product of excitation frequency and plate thickness. In the investigated case, a fundamental shear horizontal mode (SH_0_) and symmetric and antisymmetric Lamb wave modes (S_0_, A_0_) could propagate in the structure. Due to this fact and fact that each mode propagates with different phase velocities, the optimal ACT slope angle needs to be calculated for each mode separately.

## 3. Numerical Analysis

Before the experimental analysis of non-contact ACT-based elastic waves generation phase velocities for SH_0_, S_0_, and A_0_ modes for investigated composite specimen need to be determined. For this purpose, a numerical model was developed which allows determining dispersion curves. Elastic waves dispersion phenomenon is related to wavenumber *k* change with the frequency *f* of waves. Dispersion curves illustrate the dependency *k(f)*. Elastic waves dispersion curves depend on elasticity constants and the density of the material in which waves propagate. In the orthotropic laminates, like the plate considered here, material properties depend on the direction of propagation *β*. Due to this fact, it is necessary to consider dispersion curves for elastic waves at various angles of propagation *k(f, β)*. In order to determine the angle-dependent dispersion curves *k(f, β)* for investigated material, a mathematical model of the plate was developed. The numerical model is based on the modification of the semi-analytical finite element (SAFE) method proposed in [38]. The modification is based on the utilization of spectral elements instead of classic finite elements through the thickness of a laminate, preserving wave equation in the propagation direction. Due to this fact, the method is called semi-analytical spectral element (SASE) [39]. Moreover, instead of a two-dimensional approximation of cross-section of the laminate, one-dimensional four node spectral elements were utilized in the model. It has a non-uniform distribution of nodes and three degrees of freedom per node. Additionally, equations for dispersion curves are derived so that the solution can be obtained for an arbitrary angle of propagation. The general wave equation has a form of eigenvalue problem that can be solved numerically as a second-order polynomial eigenvalue problem for a given angular frequency *ω*.

(2) $$\left[A-\omega^{2}M\right]U=0,\;$$

where A—is the related to stiffness matrix and could be found in [39], M—the mass matrix, and U—the nodal displacement vector. Equation (2) could be solved numerically as a standard eigenvalue problem $\omega \left(k\right)$ (assuming given real values of wavenumbers k). Stiffness matrix components depend on an angle related to the layer orientation in the stacking sequence of a composite laminate. The SASE model was used for parametric studies of dispersion curves. The SASE model was utilized together with full wavefield measurements using SLDV and the genetic algorithm (GA) in order to obtain SASE model parameters that allow determining dispersion curves for composite material. Detailed information about this approach could be found in [39].

**Figure 2:**
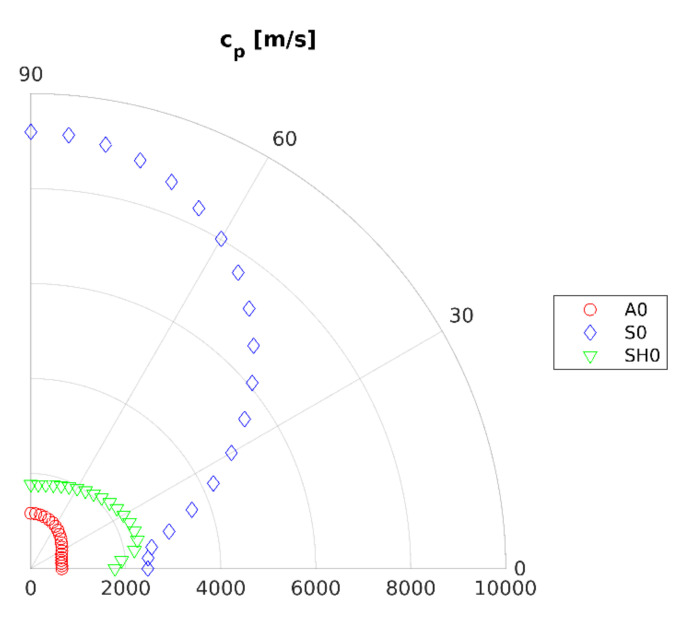
Polar plot of phase velocity for frequency 40 [kHz].

In this paper, the SASE model was developed for carbon fiber-reinforced polymer (CFRP) laminate composed of eight unidirectional reinforcing layers [0°]_8_ of a total thickness of 3.9 mm. It should be underlined here that the plate utilized in experimental research consists of 40 layers. However, due to fact that all layers are oriented at the same angle, eight layers in the numerical mode is enough to obtain good agreement with the experiment. Moreover, a reduction in the number of layers to eight allows reducing the complexity of the numerical model. Such parameters, such as phase velocity and wavenumber dependence of angle of propagation, were determined for a frequency of 40 kHz. Analyses were conducted for fundamental anti-symmetric A_0_ and symmetric S_0_ Lamb wave modes as well as for the shear horizontal SH_0_ elastic wave mode. In Figure 2, elastic wave phase velocity dependence on the angle of propagation for investigated elastic wave modes were presented.

Next, values of phase velocities for mentioned elastic wave modes in the direction along and across reinforcing fibers (0° and 90°) were extracted (Table 1).

Based on these values and Snell law (1), optimal ACT slope angles were calculated, and are presented in Table 1. Moreover, wavenumbers were extracted in order to further use in the experimental research related to dispersion curves and presented in Table 1. Further part of this research is related to experimental methods.

## 4. Experimental Setup

**Figure 3:**
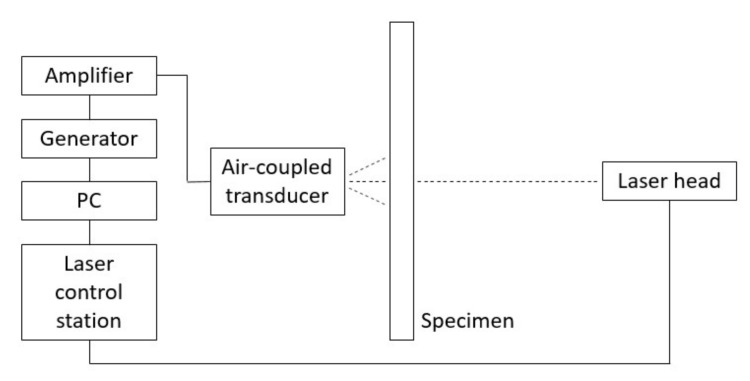
Scheme of full non-contact waves generation and sensing setup.

**Figure 4:**
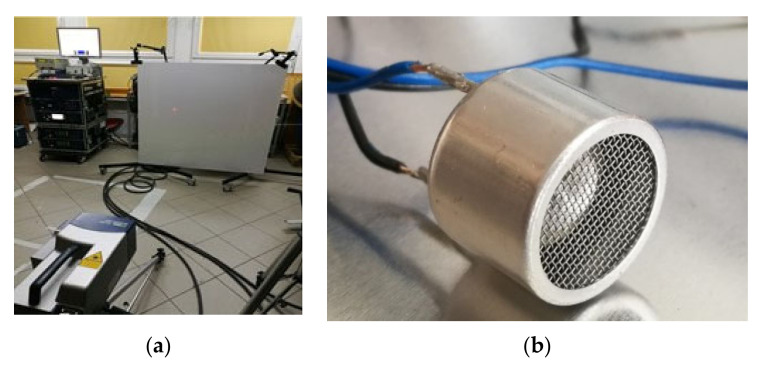
View of the measurement setup: (**a**) whole setup, (**b**) air-coupled transducer.

**Figure 5:**
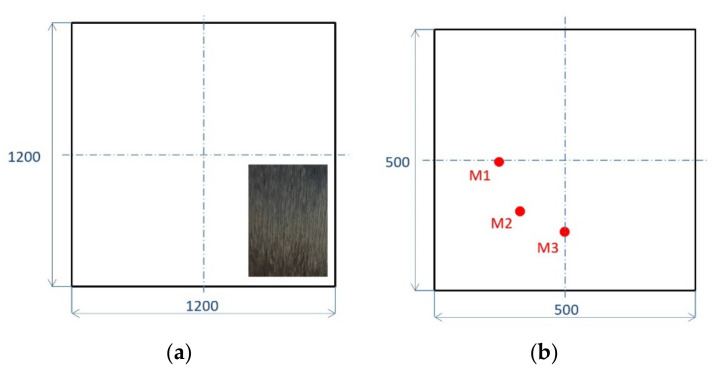
Investigated CFRP samples with different dimensions: (**a**) pristine, (**b**) with additional mass (M1–M3).

The experimental setup consisted of scanning laser Doppler vibrometer including a laser control station and a laser measurement head (Polytec PSV-400, Germany), an arbitrary signal generator, a signal amplifier, a PC computer, a composite specimen, and an air-coupled acoustic transducer ACT (Figure 3 and Figure 4). The specimens used in the research were CFRP plates with dimensions of 1200 × 1200 × 3.9 mm (pristine—Figure 5a) and 500 mm × 500 mm × 3.9 mm (with additional mass M1–M3—Figure 5b).

Both specimens consist of 40 prepreg uniaxial TDS-75 g/m^2^ layers and the IMP503Z epoxy resin. The structure of CFRP material is visible in the magnified part in Figure 5a. Moreover, the plates have a unidirectional structure—all fibers are oriented in the same direction [0°]_40_. The larger specimen was utilized in the measurements related to dispersion curves extraction and analysis of elastic waves generation using ACT. Large dimension was useful in order to avoid the influence of boundary reflected elastic waves. The smaller specimen was utilized in research related to discontinuities localization.

Research results presented in this paper were related to non-contact waves generation and sensing methods. Therefore, ACT was utilized for elastic waves generation while SLDV was utilized for waves sensing. ACT with a diameter of 16 mm, a length of 12 mm, and a base excitation frequency of 40 kHz was utilized (Figure 4b).

In one experiment instead of ACT a piezoelectric ceramic transducer in the form of a disc with a diameter of 10 mm and thickness 0.5 made of NCE51 material is utilized The aim of this experiment was to identify dispersion curves for elastic waves propagating in the plate. Only during this one measurement piezoelectric transducer, coupled with the CFRP plate (contact method), was utilized. Next, the transducer was removed from the panel surface before non-contact measurements.

Within the measurements, the plate was considered in two states—the pristine state (no discontinuities) and the damaged state (additional mass discontinuities). The specimen was mounted vertically using special holders and the whole measurement series took place in the laboratory with air conditioning, ensuring stable conditions of the air (the temperature was set to 20 ± 1 °C). The outlook of the whole measurement setup containing the plate with SLDV is shown in Figure 4a.

The first experiment was related to the identification of dispersion curves for the elastic waves propagating in the CFRP plate. For this purpose, a CFRP plate with dimension 1200 mm × 1200 mm × 3.9 mm in a pristine state (no defects) with ceramic piezoelectric transducer bonded in the middle of the plate surface was utilized. Elastic waves were excited using a piezoelectric transducer while full wavefield measurements were performed using SLDV.

Pulse excitation (broad-band) was applied to the piezoelectric transducer. The aim of this experiment was to extract dispersion curves for a wide frequency range (up to 250 kHz). Gathered full wavefield S(x,y,t) in the time–space domain is transformed to the frequency–wavenumber domain using three dimensional Fourier transform (3D FT). This allows to plot wavenumber-frequency relation (dispersion curve) for the desired angle of waves propagation.

Based on the obtained wavenumbers k, phase velocities $c_{p}$ for A_0_ and S_0_ modes were calculated for ACT frequency based on the formula:

(3) $$c_{p}=\frac{f_{ACT}}{k}$$

Then, using the Snell law (1) and calculated phase velocities for A_0_ and S_0_ modes, the optimal ACT slope angles were determined.

The next part of experimental research was related to non-contact elastic waves generation using ACT. An analysis of waves propagation in the same unidirectional CFRP plate was conducted. Elastic waves sensing was performed based on SLDV. Signal analysis was performed for three types of data. The first type was point-wise measurement data which corresponds to the elastic waves signal gathered by SLDV at a single point. The second data type was the elastic waves measurements gathered by SLDV along a line. The third type concerns full wavefield SLDV measurements where elastic waves signals were gathered in a dense mesh of measurement points.

These types of data were utilized in analysis related to the influence of different factors on elastic waves generation:

- the distance between the transducer (ACT) and the specimen (plate);
- the frequency of the excitation (in a relatively narrow range, specified later);
- the slope angle of the ACT on the effectiveness of elastic wave mode generation;
- the orientation of the reinforcing fibers (ACT direction across and along the reinforcing fibers) on the effectiveness of elastic wave mode generation and the coverage area of the plate with waves with high amplitude/energy.

Next, the experiment was conducted in order to investigate elastic waves propagation within the considered CFRP plate area and the coverage area of the plate with waves with high amplitude/energy. In this purpose, full wavefield measurements were performed.

The further step was related to analysis of elastic waves signals registered along the line. ACT was oriented at different angles from 0° (perpendicular to the plate) to 70°. Signals gathered in this manner could be presented in time–space domain $s\left(x,t\right)$. They can be next transformed to the frequency–wavenumber domain using 2D FT:

(4) $$S\left(k_{x},f\right)=\int_{-\infty }^{\infty }\int_{-\infty }^{\infty }s\left(x,t\right)e^{-j\left(2\pi ft-k_{x}\right)}dtdx,$$

where $s\left(x,t\right)$—signals in the time–space domain, $S\left(k_{x},f\right)$—the signal in the frequency–wavenumber domain, and $k_{x}$—the wavenumber along the *x* direction. Based on noticed wavenumbers in the frequency–wavenumber plots, it was possible to determine which wave modes propagate in the plate.

## 5. Experimental Results

### 5.1. Dispersion Curves

**Figure 6:**
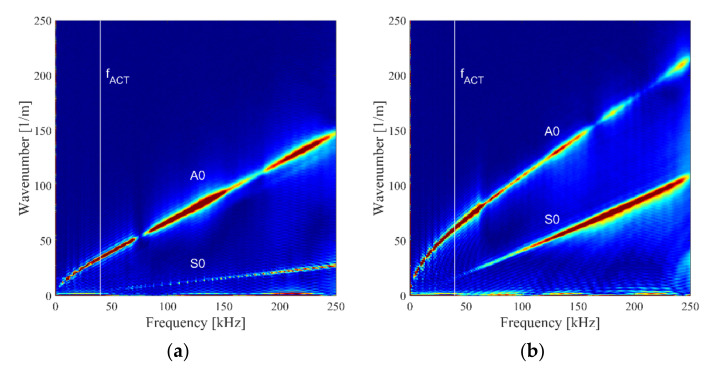
Dispersion curves for the CFRP plate for elastic waves excitation based on the piezoelectric transducer, in direction: (**a**) along fibers, (**b**) across fibers.

In this research, dispersion curves for direction along and across reinforcing fiber directions were extracted. Obtained dispersion curves for frequency up to 250 kHz based on piezoelectric transducer excitation (contact waves excitation) are depicted in Figure 6.

It should be noted that ACT works based on resonance principles and for the particular transducer used here it is 40 kHz. Therefore, the excitation frequency of ACT (40 kHz) is marked in dispersion curves with a white line in Figure 6.

That frequency was utilized in further measurements. Thus, the propagation of two elastic wave modes could be observed. By analyzing wavenumber values for 40 kHz excitation frequency and comparing with results from numerical analysis (Table 1), it could be concluded that propagation of fundamental antisymmetric A_0_ and symmetric S_0_ Lamb wave modes could be noticed. The amplitude of S_0_ wave mode was strongly reduced for frequencies below 50 kHz. The shear horizontal wave mode SH_0_ was not identified in dispersion curves for both directions (Figure 6). This could be caused by the fact that the amplitude of SH_0_ for unidirectional laminate is large at the angle of about 35° and is very small for angles along and perpendicular to reinforcing fiber direction. In this research, only one scanning head of SLDV was utilized. In this case, mainly the out-of-plane elastic waves component was measured. Therefore, there was a larger measurement sensitivity related to the A_0_ mode (dominating out-of-plane particle movement) than $S_{0}$ and SH_0_ modes where the in-plane particle movement dominated. Authors of [7] noticed only the propagation of the A_0_ wave mode in the CFRP plate.

Based on results in the form of dispersion curves (Figure 6), wavenumbers were extracted. In this case, errors related to the extraction of wavenumber from the plot were determined as ±3%. Based on the obtained wavenumbers k from dispersion curves, phase velocities $c_{p}$ for A_0_ and S_0_ and the optimal ACT slope angles were calculated. These results are given in Table 2. Errors in the case of phase velocities and ACT angles were due to errors of wavenumber estimation. Hence, in the A_0_ mode case, their values were equal to ~32° in the direction across fibers or ~16° in the direction along fibers. In the case of results for the propagation of wave across fibers, similar results were obtained for A_0_ mode for frequency 60 kHz propagating in the specimen made of aluminum with similar thickness [41]. In another cited, the paper phase velocity and ACT angle were similarly about 1100 m/s and 17°, like in our case. Authors of [42] obtained an optimal ACT angle equal to 14° for the generation of the A0 wave mode in the CFRP plate with a thickness of 2.24 mm but for the case of higher frequency (200 kHz). The CFRP plate utilized in [42] had a quasi-isotropic structure so there was only one optimal slope angle for the A_0_ wave mode which does not depend on reinforcing fiber orientation.

In the case of the S_0_ wave mode, $\theta_{opt}$ was equal to ~7° in the direction across fibers direction and ~2° in the direction along fibers. The angle for the SH_0_ mode could not be determined for measurements using wave excitation based on a piezoelectric transducer. Experimentally obtained optimal ACT slope angles for modes A_0_ and S_0_ were similar to angles obtained from numerical analysis (Table 1).

Next, numerical results from Table 1, together with experimental results from Table 2, were utilized in order to extract the optimal ACT slope angles for all modes: A_0_, SH_0_, and S_0_ for further research.

### 5.2. Non-Contact ACT-Based Elastic Waves Generation

#### 5.2.1. Point-Wise Measurements

**Figure 7:**
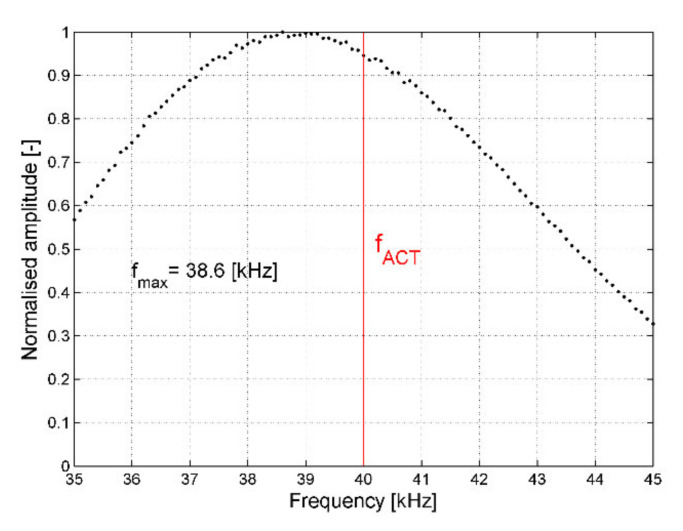
Normalized elastic waves amplitude for various excitation frequency values.

The next part of experimental research was related to non-contact elastic waves generation based on ACT and analysis of waves propagation in the pristine plate. The first considered factor impacting the waves propagation is the excitation frequency of the ACT. Although, in the case of the used ACT, the optimal excitation frequency should be equal to 40 kHz, because it is resonance frequency given by the manufacturer, tests were conducted for excitation carrier frequencies in a range of 35–45 kHz. Measurements in the single point were performed with 100-Hz steps, and the maximum amplitude of elastic waves was extracted for each frequency value and then was normalized to the unit using the largest amplitude value. In this approach, the ACT transducer was oriented perpendicularly to the CFRP plate surface and the distance between ACT and plated was equal to 24.5 mm. The results in the form of normalized elastic wave energy in a function of excitation frequency are plotted in Figure 7.

By analyzing Figure 7, one may notice that the ACT performance turned out to be the best for the frequency of ~38.6 kHz, not for 40 kHz. Signal amplitude registered for 40 kHz is ~6% lower than for 38.6 kHz. However, in the aim to let the results be comparable with other works, further measurements were conducted for an excitation frequency of 40 kHz.

**Figure 8:**
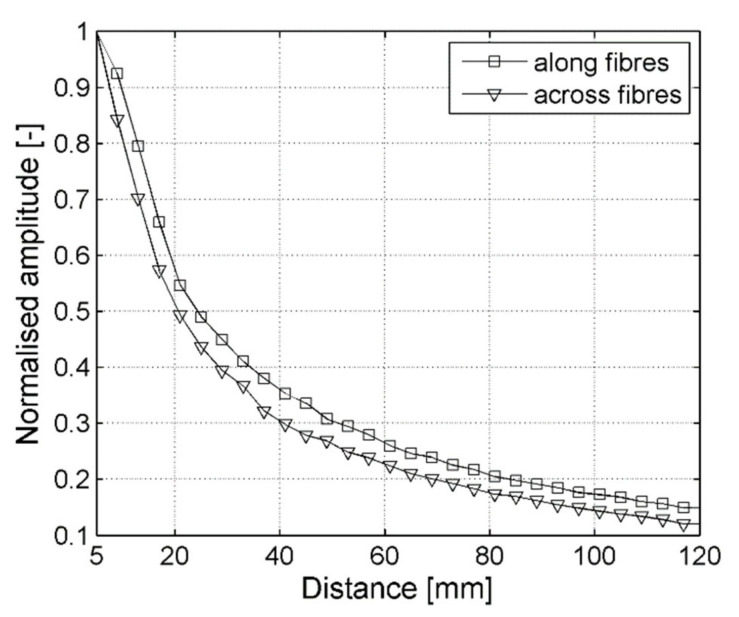
Normalized elastic waves amplitude in the direction along and across reinforcing fibers.

Another factor taken into account within point-wise measurements is the distance between the ACT and CFRP specimen surface. There were two measurement series performed for ACT direction along and across reinforcing fibers and signal sensing in one point (bellow ACT). The ACT was oriented perpendicularly to the plate surface and its distance from the plate was changed from 5 mm to 120 mm. Next, maximum signal amplitude values (vibration velocity) were extracted for each distance of ACT from the plate and normalized by maximum amplitude. Results in the form of normalized amplitudes are plotted in Figure 8.

By analyzing both graphs in Figure 8, one can observe the decrease in material particles vibration velocity with the distance between ACT and the plate. Normalized velocities registered for different distance values were similar; however, but the ACT oriented along fibers, the direction the values was slightly higher. Finally, a distance of 24.5 mm was selected for further measurements. It needs to be underlined that, if the transducer was too close to the plate, then an angular re-positioning would cause the transducer to touch the specimen [43].

#### 5.2.2. Full Wavefield Measurements

The investigated area was in the form of a square, with a side dimension equal to 420 mm in the pristine plate. ACT was located at a distance of 24.5 mm from the plate. Two cases of ACT orientation were investigated: θ = 0° (perpendicular to the plate) and θ = 30°. In the case of the angle θ = 30°, ACT was directed across and along reinforcing fibers.

**Figure 9:**
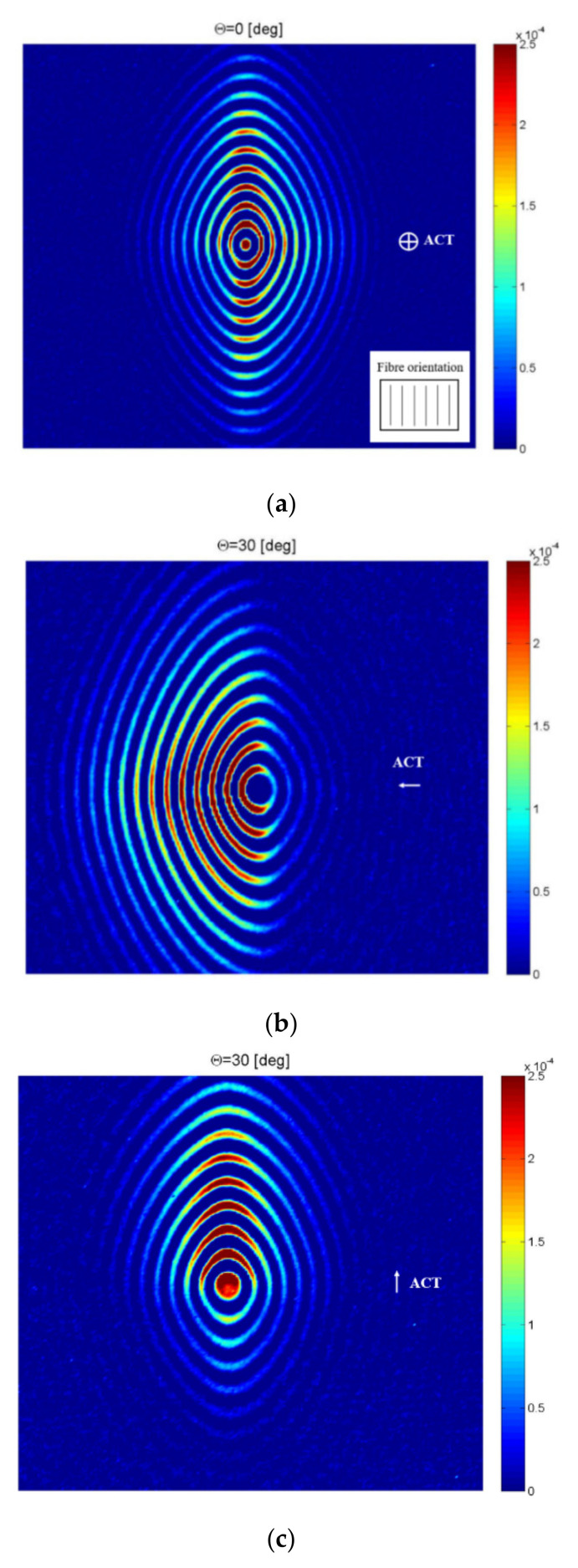
Selected frames from elastic waves propagation in the specimen for different ACT slope angles θ: (**a**) 0°, (**b**) 30° for waves generation across fibers, (**c**) 30° for waves generation along fibers.

The results in the form of selected frames from animations depicting elastic waves propagation in the plate are shown in Figure 9. The color bar scale was expressed in particle vibration velocities, measured by SLDV. The ACT direction, which was perpendicular to the plate/along or across reinforcing fibers, was marked by an arrow. Reinforcing fibers orientation was marked in Figure 9a. In the case of Figure 9a where ACT was oriented at an incidence angle θ = 0°, velocity depended on the direction of propagation and was larger in the direction along reinforcing fibers than for the direction across the fibers. The amplitude of elastic waves was also larger in the direction along the reinforcing fibers. In the case of Figure 9b, ACT was oriented at angle θ = 30° and directed across the reinforcing fibers. In this case, the amplitude of the elastic waves was larger in the direction where ACT was directed. The amplitude of the elastic waves was significantly lower in the opposite direction. In Figure 9c, ACT was oriented at the incidence angle θ = 30° and directed along the reinforcing fibers. In this case, amplitude of the elastic waves was also larger in the direction where ACT was oriented (along fibers). The amplitude of the elastic waves was significantly lower in opposite direction. It could be concluded that, in the case when ACT was oriented at the incidence angle θ = 0°, the amplitudes of elastic waves depended on material properties (reinforcing fibers direction). In the case of ACT orientation at the incidence angle θ = 30°, it was possible to control the direction where the amplitude of the generated elastic wave was larger.

This allowed for elastic waves to focus in the desired directions. Such a feature could be useful for NDT inspections of small defects. It needs to be underlined that the incidence angle θ = 30° was chosen arbitrarily in order to show the effect of the waves generation directivity.

#### 5.2.3. Determination of the Optimal Incidence Angles

Based on numerical (Section 3) and experimental results (Section 5.1) optimal ACT angles for generation of the A_0_ elastic wave mode in the direction along (17°/17°—see Table 1/Table 2) and across (32°/32°—see Table 1/Table 2) reinforcing fibers were determined.

**Figure 10:**
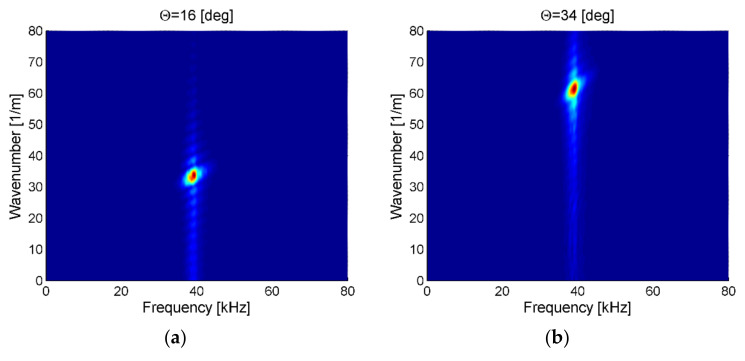
Frequency–wavenumber plot for waves generation in direction: (**a**) along fibers for θ = 16°, (**b**) across fiber for θ = 34°.

In Figure 10a, an example of the frequency–wavenumber plot for measurements in the case of ACT directed along fibers at the incidence angle θ = 16° was presented. In the case of the incidence angle θ = 16°, the maximum amplitude of elastic waves was achieved. The extracted wavenumber was equal 33.7 $\;\frac{1}{m}$ and was similar to the wavenumber for the fundamental antisymmetric Lamb wave mode A_0_ along fibers previously obtained from numerical and experimental results (see Table 1 and Table 2).

In Figure 10b, an example of the frequency–wavenumber plot for measurements for ACT directed across fibers at the incidence angle θ = 34° was presented. In the case of the incidence angle θ = 34°, the maximum amplitude of elastic waves was achieved. The extracted wavenumber was equal to 61.2 $\frac{1}{m}$ and was similar to the wavenumber for the fundamental antisymmetric Lamb wave mode A_0_ across fibers obtained previously from the numerical and experimental results (see Table 1 and Table 2).

However, it needs to be underlined that total elastic wave energy presented in frequency–wavenumber plots could be related to the energy of all wave modes that could propagate in certain plate thickness and frequency of excitation. In the investigated case according to numerical results, the Lamb wave antisymmetric $A_{0}$ and symmetric $S_{0}$ and shear horizontal ${\mathrm{SH}}_{0}$ modes could propagate in the plate. Therefore, further analysis of the frequency–wavenumber plots is needed. Such analysis was performed for the ACT slope angle value in the range of 0–70° with 1° steps. In this case cross-section of frequency–wavenumber plots for ACT, carrier excitation frequency (40 kHz) was created for each slope angle. Amplitudes were normalized to the unit using maximum amplitude.

**Figure 11:**
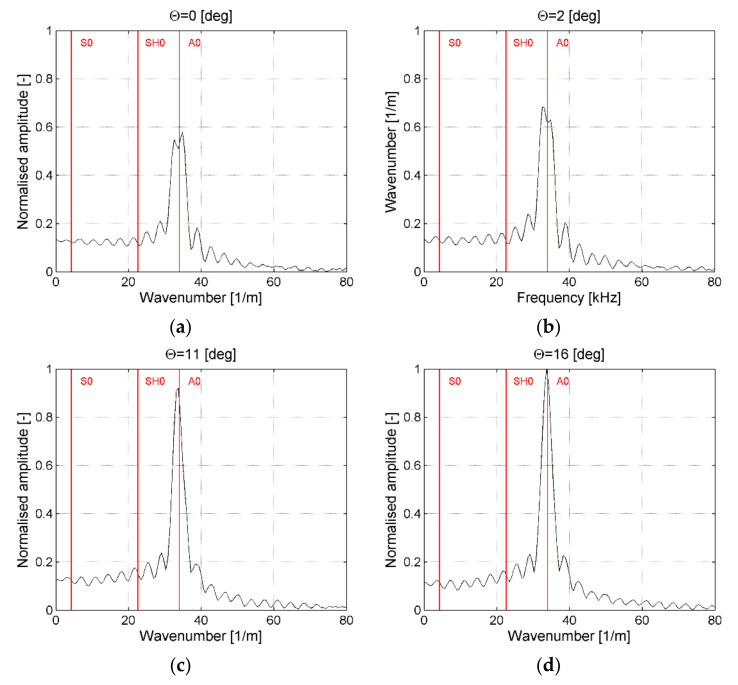
Amplitude–wavenumber plots for ACT incidence angle θ equal: (**a**) 0°, (**b**) 2°, (**c**) 11°, (**d**) 16°; waves generation along reinforcing fibers.

In Figure 11, such cross-sections of the frequency–wavenumber plot for selected ACT slope angles for the case of waves generation along the direction of reinforcing fibers are presented. In each plot, vertical lines indicate theoretical wavenumbers for S_0_, A_0_, and SH_0_ taken from numerical results (Table 1) were marked. Numerical values were presented because in the experimental results only modes S_0_ and A_0_ were observed (Table 2).

The first incidence of angle θ = 0° (Figure 11a) was related to the case when ACT was perpendicular to the plate. It could be noticed that maximum amplitude was related to the A_0_ mode extracted from the numerical model (vertical line) as well as from experimental results (Table 2). Its normalized amplitude was equal to 0.57. There was no significant energy (peaks) related to modes S_0_ and SH_0_. In the case of results in Figure 11b, ACT was oriented at the incidence angle θ = 2°, which is optimal for excitation of the S_0_ mode (Table 1 and Table 2). In this case, only the A_0_ mode was generated, but had a larger amplitude of 0.68 than in the previous case. The optimal angle for the S_0_ mode did not excite this mode. In the case of results presented in Figure 11c, ACT was oriented at the incidence angle θ = 11° which was optimal for the generation of the SH_0_ mode (Table 1). In this case, propagation of the A_0_ wave mode with an amplitude of 0.91 could be observed. There was a large number of wavenumber peaks but there was no peak that perfectly matches the wavenumber for the SH_0_ or S_0_ modes. In the case of results in Figure 11d, ACT was oriented at the incidence angle θ = 16° which was close to the optimal angle for generation of the A_0_ wave mode (Table 1 and Table 2). In this case, the largest amplitude for the A_0_ mode could be noticed and was equal to 1. In this case, a large number of peaks could be also noticed but no peak matches perfectly to S_0_ or SH_0_ wavenumber.

It could be concluded that propagation of the A_0_ wave mode was observed for all analyzed ACT slope angles and maximum amplitude of this mode was achieved for the angle θ = 16° that is similar to value from numerical and experimental results θ = 17° (Table 1 and Table 2). It should be underlined that, in the case of Figure 11a–d, there were peaks with a very small amplitude that corresponded to a wavenumber close to theoretical values of wavenumbers for the S_0_ and SH_0_ modes (Table 1). However, even if they were related to S_0_ and SH_0_ modes, its amplitudes were very small and did not dominate over the rest of visible peaks.

**Figure 12:**
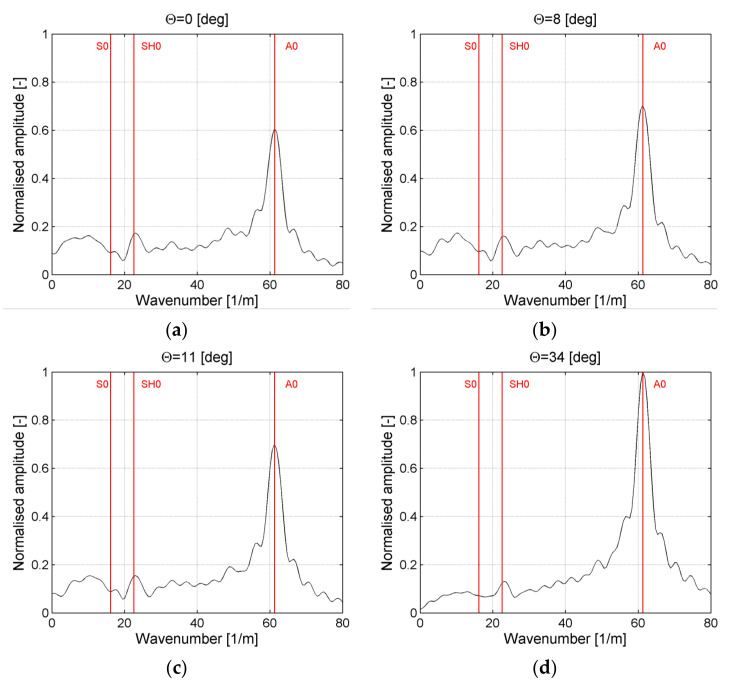
Amplitude–wavenumber plots for ACT slope angle θ equal: (**a**) 0°, (**b**) 8°, (**c**) 11°, (**d**) 34°; waves generation across reinforcing fibers.

In Figure 12, similar cross-sections of the frequency–wavenumber plot for selected ACT slope angles for the case of elastic waves generation across the direction of reinforcing fibers are presented. Results presented in Figure 12a are related to the case of ACT oriented at the incidence angle θ = 0° (perpendicular to the plate). By analyzing results Figure 12a, it could be noticed that the wavenumber peak with the largest amplitude 0.6 was related to the A_0_ mode. Moreover, a peak with an amplitude of 0.17 for wavenumber related to SH_0_ (Table 1) could be also noticed. In the case of results from Figure 12b, ACT was oriented at the incidence angle θ = 8° which was optimal for the generation of the S_0_ wave mode. Amplitude for peak related to A_0_ wavelength was equal to 0.7 but there was no peak for wavenumbers related to the S_0_ mode. Moreover, a peak with an amplitude of 0.16 for a wavenumber related to the SH_0_ mode could be distinguished in this case.

In the case of results for Figure 12c, ACT was oriented at the incidence angle θ = 11° which was optimal for the generation of the SH_0_ wave mode. The amplitude for the peak related to the wavelength of the A_0_ mode was equal to 0.7. Moreover, the peak with the amplitude of 0.155 for wavenumber related to the SH_0_ mode could be also distinguished. In the case of results from Figure 12c, ACT was oriented at the incidence angle θ = 34° which was optimal for the generation of A_0_ wave mode. In this case, the amplitude of the peak related to the wavenumber of the A_0_ wave mode was the largest and equal to 1. There was still the peak with the amplitude of 0.13 for wavenumber related to the SH_0_ mode.

It could be concluded that propagation of the A_0_ wave mode was observed for all analysed ACT slope angles and maximum amplitude of this mode was achieved for the incidence angle θ = 34° which was very close to numerical and experimental results shown in Table 1 and Table 2. Moreover, wavenumber related to the SH_0_ mode could be noticed, but, even in the case of an optimal ACT slope angle for generation of this mode, an amplitude of A_0_ modes was significantly larger than for SH_0_. These results are similar to ones obtained in [7] where authors observed that only the A_0_ wave mode propagated in a 2-mm-thick CFRP plate for a much higher frequency range—almost up to 1 MHz.

**Figure 13:**
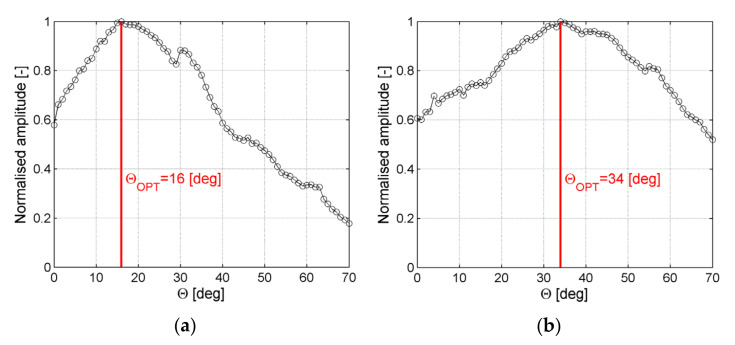
Normalized amplitude of the A_0_ wave mode in a function of ACT slope angle θ for wave generation: (**a**) along reinforcing fibers, (**b**) across reinforcing fibers.

In Figure 13, amplitudes for A_0_ wave mode taken from frequency–wavenumber plot cross-sections for the ACT slope angles θ from 0° to 70° with step 1° for waves generation in the direction along and across fibers are plotted. Amplitudes were normalized to the unit using the largest amplitude achieved for optimal ACT slope angles.

In Figure 13a amplitudes for the A_0_ mode in a function of ACT orientation angle for the case of waves generation along reinforcing fibers are plotted. It could be noticed largest amplitude was achieved for the incidence angle θ_OPT_ = 16°. In Figure 13b amplitudes for the A_0_ mode in a function of ACT orientation angle for the case of waves generation across reinforcing fibers are plotted. It could be noticed that the largest amplitude was achieved for the incidence angle θ_OPT_ = 34°.

**Figure 14:**
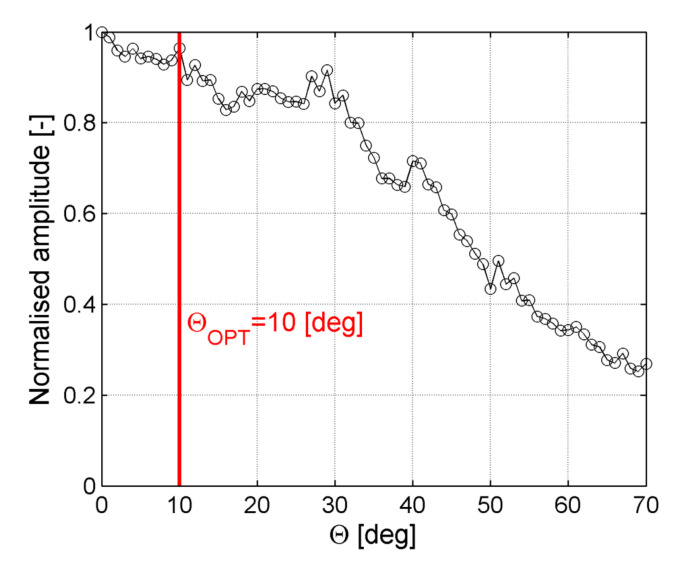
Normalized amplitude of the SH_0_ wave mode in a function of ACT slope angle θ for waves generation across reinforcing fibers.

In Figure 14 amplitudes for the SH_0_ mode in a function of ACT orientation angle for the case of waves generation across reinforcing fibers are plotted. It could be noticed that the largest amplitude was achieved for the incidence angle θ = 0° and then the local maximum for θ_OPT_ = 10° which is close to the value obtained from numerical and experimental results (Table 1 and Table 2). However, the amplitude of this mode was still significantly lower than the amplitude of the A_0_ mode (see Figure 12). This problem still needs to be further investigated.

In Table 3, a summary of extracted optimal ACT slope angles based on different methods is given. The first method was based on numerical SASE. The second was the experimental method related to dispersion curves identification (based on piezoelectric wideband contact waves generation). The third was the experimental method based on non-contact ACT-based waves generation.

In the case of numerical results from the SASE method, propagation of A_0_, S_0_, and SH_0_ was taken into account, and optimal ACT angles were extracted for these modes. In the case of experimental results based on contact waves generation (piezoelectric transducer), propagation of only A_0_ and S_0_ wave modes was noticed. In the case of ACT slope angles for non-contact waves generation (ACT), an error equal to ±1° was assumed. This error results from the scale in the utilized protractor (1°) used for slope angle set. In the ACT-based results, only the A_0_ wave mode was noticed in the direction along fibers but A_0_ and SH_0_ modes were observed in the direction across the fibers.

Concluding this section, it is possible to efficiently generate an A_0_ wave mode using a non-contact method based on ACT. There is an optimal ACT slope angle that helps to achieve the largest amplitude of the A_0_ mode. The optimal incidence angle depends on waves generation direction (along/across reinforcing fibers). It is also possible to generate the A_0_ mode with ACT oriented perpendicularly to the plate, but the amplitude of the A_0_ wave mode is smaller (~60% of value for optimal angle). In the case of ACT oriented perpendicularly to the plate, waves propagate in all directions (amplitude depends on fiber orientation). In the case of ACT oriented at optimal incidence angles, guided waves are focused in the direction related to ACT. In this case, the whole surface is not covered by elastic waves, but it is possible to focus elastic waves with high amplitudes in the desired direction. This could be observed for the ACT angle θ = 30° in Figure 9b,c. This phenomenon of waves focusing could be helpful in the detection of small defects.

## 6. Discontinuity Localization

Due to the fact that A_0_ is an efficiently generated elastic wave mode, further research presented in this paper is related only to this mode. After verification of optimal ACT slope angles ensuring the efficient generation of A_0_ waves mode, it is desired to analyze elastic waves propagation in the plate with discontinuities simulating damage. The investigated plate has the dimensions of 500 mm × 500 mm × 3.9 mm and was made out of the same material as the plate in the previous analysis. Discontinuities in the form of three additional masses (mass ~15 g) bonded to the plate surface, thereby causing utilization of the local plate thickness, stiffness, and mass change. This is a very simple method of damage simulation performed in order to avoid permanently damaging the plate. However, the real damage is also related to stiffness and thickness change (e.g., delamination).

Full wavefield measurements were performed using the same equipment for the measurements of the plate in a pristine state. Elastic waves generation was based on the ACT oriented perpendicularly to the plate surface and at optimal θ values. Based on full wavefield measurement, RMS wave energy was calculated based on the formula:

(5) $$\mathrm{RMS}\left[i,j\right]=\sqrt{\frac{1}{N}\sum_{k=1}^{N}s{\left[i,j,k\right]}^{2},}$$

where $\mathrm{RMS}\left[i,j\right]$—is the wave energy at the point with indexes i and j, $s\left[i,j,k\right]$—the signal taken at the point with indexes i and j, k—is the index of sample, and N—is the total number of samples in the signal.

**Figure 15:**
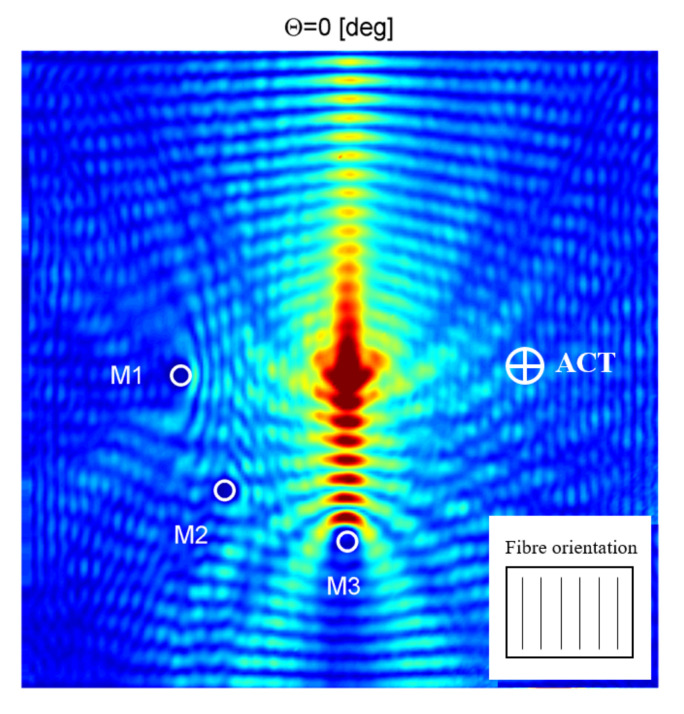
RMS energy map for the plate with discontinuities; ACT oriented at θ = 0° (perpendicular to the plate).

Next, results in form of RMS wave energy maps were prepared. In Figure 15, the example of RMS wave energy map for the case when ACT orientation is perpendicular to the plate surface (θ = 0°) is presented. Additional masses were marked as M1–M3 and ACT orientation was marked by an arrow. Moreover, reinforcing fibers orientation is indicated in Figure 15.

By analyzing the results presented in Figure 15, it could be noticed that elastic wave energy is concentrated in the middle of the plate where the acoustic waves from ACT is converted to elastic waves in the plate. Elastic wave energy is focused along the direction of reinforcing fibers. Additional masses, M1 and M3, were detected easily by observing the concentration of wave energy near additional masses and wave energy reduction behind masses. There are only very small changes in elastic wave energy distribution around the mass M2. Such slope angle of ACT (θ = 0°) was utilized in the work [44]. Such angle makes the measurement setup much simpler in which two ACTs are used in the transmission mode and pulse-echo. In our case where waves are excited by ACT and sensed by SLDV, ACT does not have to be set at angle θ = 0° (perpendicular to the sample).

**Figure 16:**
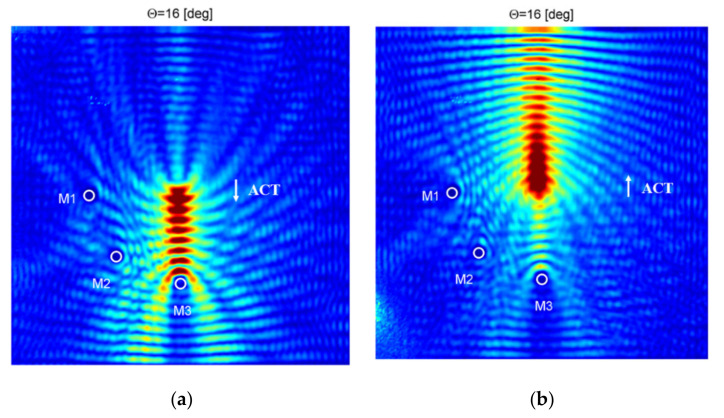
RMS energy map for the plate with discontinuities; ACT oriented at θ = 16° (along reinforcing fibers) and directed to: (**a**) bottom part of the plate, (**b**) the top part of the plate.

In Figure 16, an RMS energy map for the case when ACT is oriented at the optimal angle θ = 16° for the case of the A_0_ wave mode generation in the direction along reinforcing fibers is presented. In Figure 16a, RMS energy map for ACT directed in the bottom part of the plate is presented. Results are similar to the previous case. Wave energy is focused in direction of ACT (bottom part of the plate) and along the fibers. In this case, half the area of the considered plate is better covered by elastic waves (the area where elastic waves propagate with relatively high amplitudes is higher). Additional masses M1–M3 could be localized. A change in the elastic wave energy caused by discontinuity M3 is the largest. In Figure 16b, an RMS energy map for ACT directed in the top part of the plate is presented. Wave energy is focused in direction of ACT (the bottom part of the plate) and along the reinforcing fibers. Additional masses M1–M3 could be localized. It is interesting that discontinuity M3 could still be very easily localized besides the fact that ACT is directed in opposite direction (elastic wave energy is focused on the opposite part of the plate than M3 is located).

**Figure 17:**
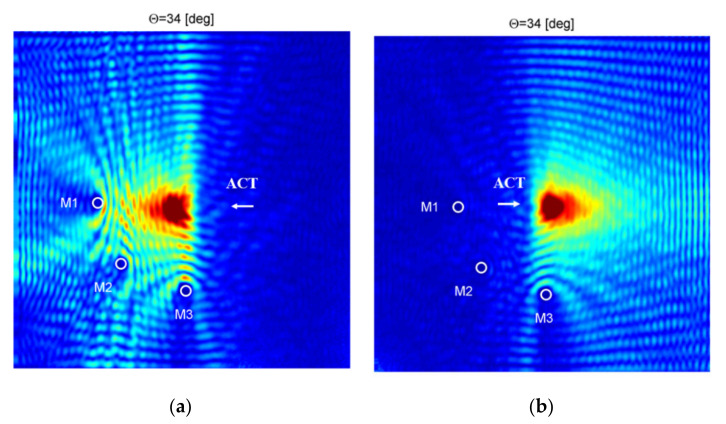
RMS energy map for the plate with discontinuities; ACT oriented at θ = 34° (across reinforcing fibers) and directed to: (**a**) left part of plate, (**b**) right part of plate.

In Figure 17, RMS wave energy map for the case when ACT is oriented at the optimal angle θ = 34° for the case of the A_0_ wave mode generation in the direction across reinforcing fibers. In Figure 17a, an RMS energy map for ACT directed in the left part of the plate is presented. Wave energy is focused in the direction of ACT (the left part of the plate). Additional mass M1–M3 could be easily localized. In this case, half of the considered plate area (on the left) is better covered by elastic waves, i.e., the area where elastic waves propagate with relatively high signal amplitudes is higher. In Figure 17b, an RMS energy map for ACT directed in the right part of the plate is presented. Wave energy is focused in the direction of ACT (the right part of the plate). In this case, only discontinuity M3 could still be localized. In this case, when waves are generated and propagate across fibers, larger damping of the elastic wave mode A_0_ could be noticed (see the right part of the plate without discontinuity in Figure 17b) than in the case of waves generation and propagation along fibers (see the top part of the plate without discontinuity in Figure 16a).

By analyzing results in Figure 16 and Figure 17, it can be found that ACT still offers good detection ability, though it seems it is easier to detect defects when they are located in direction of the ACT.

In this case of ACT orientation perpendicular to the plate (θ = 0°), the highest coverage area of the plate by elastic waves can be achieved. Nevertheless, the amplitude of wave energy is smaller than for the optimal ACT angle. The optimal ACT slope angle helps to generate waves with a higher amplitude and to focus the wave in the desired direction. It should be underlined that an additional focusing technique could improve this effect. For example, a passive ACT focusing technique could be utilized in which special focusing arrangement in the form of a Newtonian–Cassegrain design is applied [45]. Moreover, by using ACT with an optimal slope angle, it is possible to take advantage of elastic waves focused and directed on the defect from different sides [42].

## 7. Conclusions

Results showed that there are optimal ACT slope angles that help to effectively generate the A_0_ wave mode. Due to the stack sequence in the composite plate (unidirectional composite), ACT slope angles are different for waves generation in the direction along and across reinforcing fibers direction. In the case of across fiber direction, it was also possible to excite the SH_0_ wave mode but with a very small amplitude (amplitude of SH_0_ is equal to ~0.22 amplitude of the A_0_ mode).

The air-coupled transducer oriented perpendicularly to the plate is an omnidirectional source of generated elastic waves. The ACT for optimal slope angles for the A_0_ mode is not the omnidirectional source of elastic waves. However, in the case of optimal angles, elastic waves amplitudes are almost two times higher. This helps to focus the elastic wave energy in the chosen direction.

For the purpose of an optimal ACT slope angle, a numerical method based on the SASE model was utilized. The aim of this method was to extract the ACT angles based on phase velocities, and Snell law dispersion curves were also estimated experimentally by using broadband excitation applied to the piezoelectric transducer and sensing by the SLDV.

The distance between ACT and the investigated specimen (plate) surface has a great impact on the amplitudes of registered signals. Based on the current investigation, keeping the distance as low as possible is recommended.

The frequency of excitation referred to the used transducer also has a significant impact on signal amplitudes of elastic waves propagating in the structure. After the analysis, it turned out that the best performance of the used ACT was for the frequency of 38.6 kHz, not 40 kHz.

Presented results showed that a frequency of 40 kHz and ACT-based non-contact waves generation, together with SLDV-based waves sensing, helped to detect investigated additional masses in the CFRP plate.

Further research will be related to higher frequencies, more excitation sources, and the problem of S_0_ and SH_0_ modes generation. Moreover, a problem of damage extent estimation will be investigated in further research.

## Figures and Tables

**Table 1:** Numerical results.

Wave Mode	Wavenumber [1/m]	Phase Velocity [m/s]	Optimal ACT Angle θ [deg]
direction along fibres			
A_0_	34.0	1176.5	16.9
S_0_	4.3	9302.3	2.1
SH_0_	22.58	1771.5	11.2
direction across fibres			
A_0_	61.3	652.52	31.7
S_0_	16.24	2461.31	8
SH_0_	22.58	1771.5	11.2

**Table 2:** Experimental results.

Wave Mode	Wavenumber [1/m]	Phase Velocity [m/s]	Optimal ACT Angle [deg]
direction along fibres			
A_0_	34.2 ± 1	1169.6 ± 33	17 ± 0.5
S_0_	4.4 ± 0.13	9091 ± 261	2.1 ± 0.05
direction across fibres			
A_0_	61.2 ± 1.8	653.59 ± 19	31.6 ± 1
S_0_	14.7 ± 0.4	2706.4 ± 72	7.2 ± 0.2

**Table 3:** Optimal ACT slope angles for different methods.

Wave Mode	Optimal ACT Slope Angle [deg]		
	Numerical Results	PZT Measurements	ACT Measurements
direction along fibres			
A_0_	16.9	17 ± 0.5	16 ± 1
S_0_	2.1	2.1 ± 0.05	-
SH_0_	11.2	-	-
direction across fibres			
A_0_	31.7	31.6 ± 1	34 ± 1
S_0_	8	7.2 ± 0.2	-
SH_0_	11.2	-	10 ± 1

## Data Availability

The data presented in this study are available on request from the corresponding author. The data are not publicly available at this time as the data also forms part of an ongoing study.
